# ADAPTATION OF THE E-PAINSUPPORT INTERVENTION FOR ADRD CAREGIVERS

**DOI:** 10.1093/geroni/igae098.0931

**Published:** 2024-12-31

**Authors:** Masako Mayahara

**Affiliations:** Washington University in St. Louis, St. Louis, Missouri, United States

## Abstract

The lack of adherence by family caregivers to prescribed analgesic regimens has been recognized as a significant barrier to effective pain management in hospice. Advancements in healthcare technology provide an opportunity to enhance timely access to pain education and promote better adherence to analgesic regimens for family caregivers of patients with Alzheimer’s Disease and Related Dementias (ADRD). The aims of this presentation are to report the findings from a recently completed randomized controlled trial of hospice patient-caregiver dyads and discuss adaption of the intervention for ADRD caregivers. The initial efficacy trial used a 2-group, 2-week longitudinal, randomized controlled trial design. The study participants were recruited from four hospice agencies located in in the Midwest. Forty-four patient-caregiver dyads participated. There was a small positive effect size (d=0.27) associated with the e-PainSupport intervention, suggesting efficacy in reducing pain intensity. The regression analysis revealed that increased knowledge had a significant independent effect on decreasing pain (p<0.01). Female caregivers and children of patients were more likely to respond to the intervention compared male or spouse caregivers. The study highlighted the importance of caregiver knowledge in reducing patient pain. Adaptation of the intervention for ADRD caregivers requires a tailored approach to increase usability for male and spouse caregivers.

